# Automated Quantitative Susceptibility and Morphometry MR Study: Feasibility and Interrelation Between Clinical Score, Lesion Load, Deep Grey Matter and Normal-Appearing White Matter in Multiple Sclerosis

**DOI:** 10.3390/diagnostics14232669

**Published:** 2024-11-27

**Authors:** Gibran Manasseh, Tom Hilbert, Mário João Fartaria, Jeremy Deverdun, Meritxell Bach Cuadra, Bénédicte Maréchal, Tobias Kober, Vincent Dunet

**Affiliations:** 1 Department of Diagnostic and Interventional Radiology, Lausanne University Hospital and University of Lausanne, 1011 Lausanne, Switzerland; gibran.manasseh@chuv.ch (G.M.); tom.hilbert@siemens-healthineers.com (T.H.); mario.fartaria_de_oliveira@siemens-healthineers.com (M.J.F.); meritxell.bachcuadra@unil.ch (M.B.C.); benedicte.marechal@siemens-healthineers.com (B.M.); tobias.kober@siemens-healthineers.com (T.K.); 2 Advanced Clinical Imaging Technology, Siemens Healthcare AG, 1015 Lausanne, Switzerland; 3 Signal Processing Laboratory (LTS5), École Polytechnique Fédérale de Lausanne (EPFL), 1015 Lausanne, Switzerland; 4 I2FH, Institut d’Imagerie Fonctionnelle Humaine, Montpellier University Hospital Center, Gui de Chauliac Hospital, 34295 Montpellier, France; jeremy.deverdun@neuf.fr; 5 CIBM Center of Biomedical Imaging, 1015 Lausanne, Switzerland

**Keywords:** multiple sclerosis, quantitative susceptibility mapping, morphometry, atrophy, clinical disability, EDSS

## Abstract

Introduction: Lesion load (LL), deep gray matter (DGM) and normal-appearing white matter (NAWM) susceptibility and morphometry may help in monitoring brain changes in multiple sclerosis (MS) patients. We aimed at evaluating the feasibility of a fully automated segmentation and the potential interrelation between these biomarkers and clinical disability. Methods: Sixty-six patients with brain MRIs and clinical evaluations (Expanded Disability Status Scale [EDSS]) were retrospectively included. Automated prototypes were used for the segmentation and morphometry of brain regions (MorphoBox) and MS lesions (LeManPV). Susceptibility maps were estimated using standard post-processing (RESHARP and TVSB). Spearman’s rho was computed to evaluate the interrelation between biomarkers and EDSS. Results: We found (i) anticorrelations between the LL and right thalamus susceptibility (rho = −0.46, *p* < 0.001) and between the LL and NAWM susceptibility (rho = [−0.68 to −0.25], *p* ≤ 0.05); (ii) an anticorrelation between LL and DGM (rho = [−0.71 to −0.36], *p* < 0.04) and WM morphometry (rho = [−0.64 to −0.28], *p* ≤ 0.01); and (iii) a positive correlation between EDSS and LL (rho = [0.28 to 0.5], *p* ≤ 0.03) and anticorrelation between EDSS and NAWM susceptibility (rho = [−0.29 to −0.38], *p* < 0.014). Conclusions: Fully automated brain morphometry and susceptibility monitoring is feasible in MS patients. The lesion load, thalamus and NAWM susceptibility values and trophicity are interrelated and correlate with disability.

## 1. Introduction

Multiple sclerosis (MS) is a chronic demyelinating disease that affects both the gray and white matter of the brain. While MRI is a valuable tool to diagnose and monitor MS, conventional MRI techniques fail to correlate accurately the lesion load with clinical disability, possibly in part due to disease activity beyond MR-visible lesions [1]. Novel MRI techniques are therefore needed to better characterize the total MS burden in the brain and to improve treatment monitoring. Quantitative susceptibility mapping (QSM) is an emerging technique that can potentially facilitate the characterization of inflammation and demyelination in the brain of MS patients [2,3]. In MS, myelin and iron content changes are thought to be the main factors responsible for susceptibility changes inside and outside the visible lesions [4,5]. Thus, QSM is increasingly used for the characterization of iron load in the deep gray matter (DGM), as well as in lesions and normal-appearing white matter (NAWM) [6,7]. Among others, changes in DGM susceptibility can be observed in cases of clinically isolated syndrome (CIS) [8]. Magnetic susceptibility in specific brain regions and lesions might hence be a potential biomarker to monitor disease progression. However, the interrelation between susceptibility in the brain and the disease state is still not widely investigated. Previous studies have shown higher susceptibility for basal ganglia (probably due to demyelination) and lower susceptibility for thalamus and NAWM (probably due to iron loss) with disease progression [7,9,10]. Moreover, thalamic damage seems to be related to NAWM damage [11]. However, because of technical differences, studies are not fully comparable and conclusive.

Moreover, there is increasing evidence that brain atrophy might be a stronger biomarker than lesion load for predicting progressing disability. DGM atrophy, specifically of the thalamus, seems to have the strongest association with MS clinical burden [12,13,14,15,16]. For instance, it has been shown that atrophy is present in CIS patients at presentation, particularly in the thalamus, and other DGM structures [17]. Also, based on tractography analysis, it seems that white matter lesions are likely causing upstream and downstream degeneration and a subsequent reduction in thalamic volume in patients with MS [16,18].

Analyzing lesion load, atrophy, susceptibility and clinical disability simultaneously is important because of their potential interrelation as well as direct influence. For instance, it is known that local susceptibility can change with iron content, but also with local atrophy [19]. To our knowledge, there is no study exploring the interrelation between lesion burden and DGM or NAWM susceptibility and trophicity in MS in the same cohort. Before analyzing such interrelations under the scope of clinical data for disease burden assessment and monitoring, the feasibility of an automated and reproducible method should be established.

The main goal of this study was thus to assess the feasibility of a fully automated quantitative segmentation method. Second, we aimed at evaluating the potential interrelation between lesion load and susceptibility and morphometry in DGM and NAWM and between NAWM susceptibility and DGM susceptibility. Finally, we evaluated the interrelation between these imaging markers and individual clinical disability.

## 2. Materials and Methods

### 2.1. Study Protocol

This retrospective observational single-center study was conducted according to the STROBE guidelines [20]. Patients with MS who were referred to our institution for brain imaging between October 2016 and July 2017 were screened. Inclusion criteria were (a) being diagnosed with multiple sclerosis according to the 2010 McDonald criteria [21] and (b) having underwent at least one brain MRI with gadolinated contrast media administration and the acquisition protocol described below. Except for age and sex available on DICOM tags, no further clinical data was recorded. Also, all collected imaging data were anonymized to comply with national ethical guidelines. Institutional Review Board approval (CER-VD 2023-01584) and patient consent were obtained according to the Swiss Federal Act on Research involving Human Beings from 2011 (HRA, Art. 3), and the study was conducted in accordance with the World Medical Association Declaration of Helsinki. Patient clinical disability was assessed using the Expanded Disability Status Scale (EDSS) at the time of MRI, which was retrieved from patients’ hospital records.

### 2.2. MRI Acquisition

All patients were imaged on a 3T scanner (MAGNETOM Skyra, Siemens Healthcare, Erlangen, Germany). The acquisition protocol included 3D FLAIR, unenhanced T1w MP-RAGE and double-echo (TE = 20/40 ms) gradient echo susceptibility weighted imaging (SWI) sequences. Acquisition parameters are given in Table 1.

### 2.3. MRI Post-Processing

The segmentation of MS lesions was performed using a fully automated prototype method LeManPV [22,23,24], which takes as input 3D FLAIR and MP-RAGE images. Brain lobes and segmentations of WM, thalamus and basal ganglia (putamen, pallidum and caudate) were obtained using the MorphoBox prototype [25], taking unenhanced MP-RAGE images as input. NAWM was defined as the volume of the remaining segmented WM after subtracting the MS lesion volume in WM. Lesion load was defined as the volume in milliliters of all the lesions present in a given region, as it was derived from the binary masks from the automated lesion segmentation. The age- and gender-corrected Z-scores (i.e., how many standard deviations is this volume away from the mean volume in a healthy population) of the DGM regions were used for morphometric analysis.

**Figure 1:**
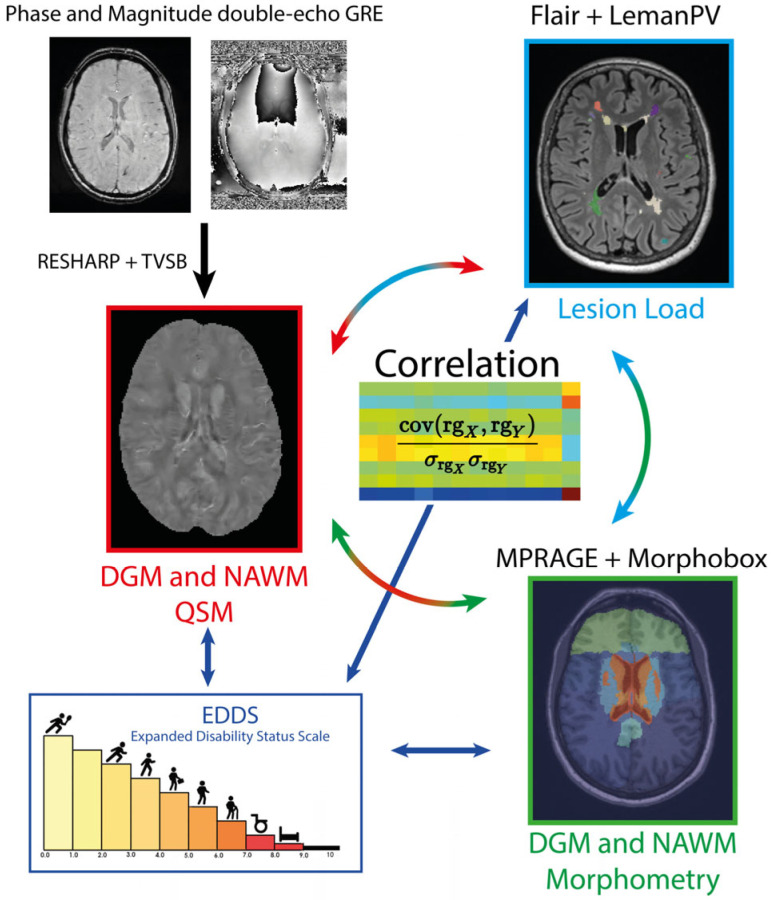
Imaging processing. Schematic representation of algorithms used to compute QSM using Regularization Enabled Sophisticated Harmonic Artifact Reduction for Phase data (RESHARP) and the Total Variation using Split Bregman (TVSB) on double-echo gradient echo (GRE) sequences, extract MS lesions using LeManPV from 3D FLAIR data and extract brain regions using Morphobox from 3D unenhanced T1-MP-RAGE data. Correlations between results and with the EDSS were then computed.

QSM maps were estimated from phase and magnitude SWI data. The computation was performed using an in-house dedicated Matlab SPM Toolbox, which integrates a standard post-processing pipeline that implements the Regularization Enabled Sophisticated Harmonic Artifact Reduction for Phase data (RESHARP) and the Total Variation using Split Bregman (TVSB) algorithms [26,27]. This Matlab SPM Toolbox can be shared upon request. Since QSM reconstruction constitutes an ill-posed inverse problem, it is only possible to quantify magnetic susceptibility in relation to a reference value rather than in absolute terms [28,29]. To account for this offset, the median QSM value of the whole brain was subtracted from each extracted QSM value of the same patient, as previously reported [30]. The MP-RAGE images where rigidly registered to the SWI images using ELASTIX [31]. The resulting transformation matrices where then used to transform the LeMan-PV and MorphoBox output masks into the QSM space. To mitigate the effect of partial volume between volumes, all binary masks were eroded by one voxel. Subsequently, median QSM values of thalamus, basal ganglia and NAWM were extracted (see Figure 1). The median was preferred over the mean because QSM values were not normally distributed within the regions and because of its robustness to outliers (e.g., due to inaccurate segmentation of volumes).

### 2.4. Statistical Analysis

All statistics were performed with Matlab R2018b (The MathWorks Inc., Natick, MA, USA). Continuous variables are reported as median and interquartile range (IQR). Categorical variables are reported as number or percentage. Spearman’s correlation coefficients (rho) were computed to evaluate potential interrelation between (i) lesion load and DGM magnetic susceptibility and morphometry; (ii) lesion load and NAWM magnetic susceptibility and lesion load and total WM morphometry; and (iii) lesion load and DGM and NAWM morphometric and susceptibility values with the EDSS (Figure 1). To account for possible age-related susceptibility variations, the correlation coefficients were controlled by age using partial correlation [32]. To account for multiple comparisons, the threshold for significant *p*-values was corrected using the False Discovery Rate (FDR) procedure [33].

## 3. Results

### 3.1. Study Population

Overall, a total of 66 MS patients (50 women, median age = 38 years, IQR = 14 years, range 18–66 years) consecutively acquired in daily practice over 2 years were retrospectively enrolled. From the entire cohort, five patients were excluded due to poor image quality related to motion or magnetic artifacts. Complete post-processing was thus successful in 93.2% of patients. The whole processing (FLAIR, T1 MP-RAGE and QSM) was performed in 10 min per patient. Overall, patients had 1019 non-enhancing lesions. Fourteen patients (20%) additionally had a total of 37 enhancing lesions (range 1–10), with their size varying between 14 and 320 μL. Enhancing lesions thus represent 3.6% of all the lesions. Removing those fourteen patients from the analysis presented below did not change the results. At the time of the study, some of the patients were undergoing standard pharmacological treatments for multiple sclerosis, while 11 patients remained untreated. The different treatments administered to the 61 patients included in the analysis are listed in Table 2.

### 3.2. Interrelation Between Lesion Load, DGM Susceptibility and DGM Morphometry

**Figure 2:**
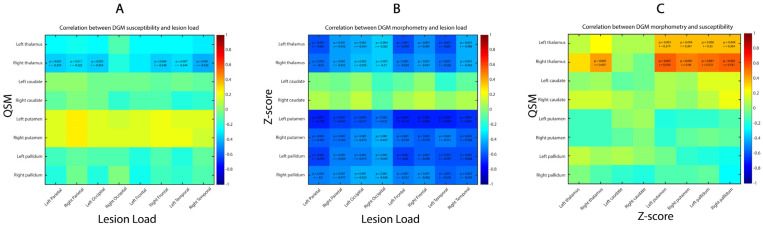
Correlation between lesion load, DGM susceptibility and morphometry. (**A**) Interrelation between lesion load (abscissa) and DGM susceptibility (ordinate). (**B**) Interrelation between lesion load (abscissa) and DGM morphometry (ordinate). (**C**) Interrelation between DGM morphometry (abscissa) and susceptibility (ordinate). Only correlation coefficients with significant *p*-values are written down, with threshold value corrected for multiple comparison.

The susceptibility values and Z-scores of the thalamus, basal ganglia and NAWM are displayed in Table 3. Right thalamus susceptibility was negatively correlated with the total lesion load (rho = −0.43, *p* < 0.001), as well as with each individual lobar lesion load except the right occipital and left frontal lobes (Figure 2A). The left thalamus, left and right putamen, left and right caudate, and left and right pallidum did not show any significant correlation with lesion load.

We found moderate to strong significant negative correlations between lesion load and DGM morphometry, except for the caudate nuclei (Figure 2B). The strongest correlation was between left putamen Z-score and left frontal lobe (rho = −0.71, *p* < 0.001) and the weakest correlation between the left thalamus and the right occipital lobe (rho = −0.36, *p* = 0.004).

We found only positive significant correlations between thalamus susceptibility values and thalamus, putamen and pallidum DGM morphometric values (Figure 2C). The strongest correlation was between right thalamus susceptibility and left putamen Z-score (rho = 0.56, *p*-value < 0.0001).

### 3.3. Interrelation Between Lesion Load, NAWM Susceptibility and WM Morphometry

**Figure 3:**
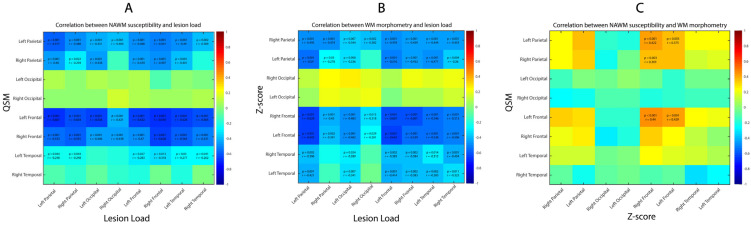
Correlation between lesion load, NAWM susceptibility and WM morphometry. (**A**) Interrelation between lesion load (abscissa) and NAWM susceptibility (ordinate). (**B**) Interrelation between lesion load (abscissa) and WM morphometry (ordinate). (**C**) Interrelation between WM morphometry (abscissa) and NAWM susceptibility (ordinate). Only correlation coefficients with significant *p*-values are written down, with threshold value corrected for multiple comparison.

We found weak to strong negative correlations between lobar and total lesion load with the magnetic susceptibility of the NAWM in the frontal and parietal lobes and left temporal lobe (rho = [−0.68 to −0.25], all *p* ≤ 0.05) (Figure 3A). No correlation was found for right temporal and bilateral occipital NAWM magnetic susceptibility. The median magnetic susceptibility of the whole NAWM was negatively correlated with lobar and total lesion load (rho = [−0.71 to −0.46], all *p* < 0.001).

We found weak to strong negative correlations between lobar and total lesion load and the morphometry of the WM in each individual lobe except right and left occipital lobes (rho = [−0.64 to −0.28], all *p* ≤ 0.01, Figure 3B). The morphometry of the total WM was negatively correlated with the lesion load in left parietal, both frontal, both temporal lobes and with the total lesion load (rho = [−0.42 to −0.31], all *p* ≤ 0.01).

We found weak to moderate positive correlation between WM morphometry and NAWM susceptibility values between frontal lobe morphometric values and left frontal and both parietal lobe susceptibility (rho = [0.37 to 0.44], all *p* ≤ 0.01, while no correlation was found in the temporal and occipital lobes (Figure 3C).

### 3.4. Interrelation Between NAWM and DGM Susceptibility

**Figure 4:**
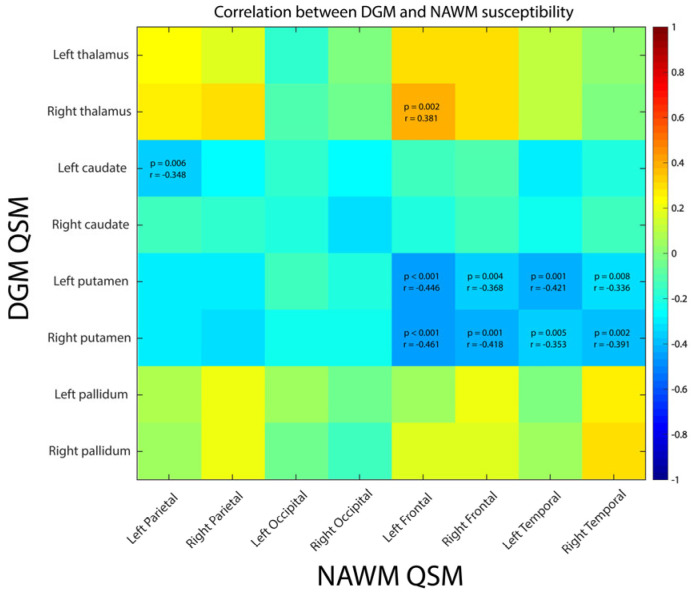
Correlation between DGM and NAWM susceptibilities. Only correlation coefficients with significant *p*-values are written down, with threshold value corrected for multiple comparison.

Left and right putamen susceptibility values were negatively moderately correlated with fronto-temporal and total NAWM median susceptibility (rho = [−0.46 to −0.34], *p* ≤ 0.008, Figure 4). No systematic correlation was found for thalamus, pallidum and caudate.

### 3.5. Interrelation Between EDSS Clinical Score and MRI Metrics

**Figure 5:**
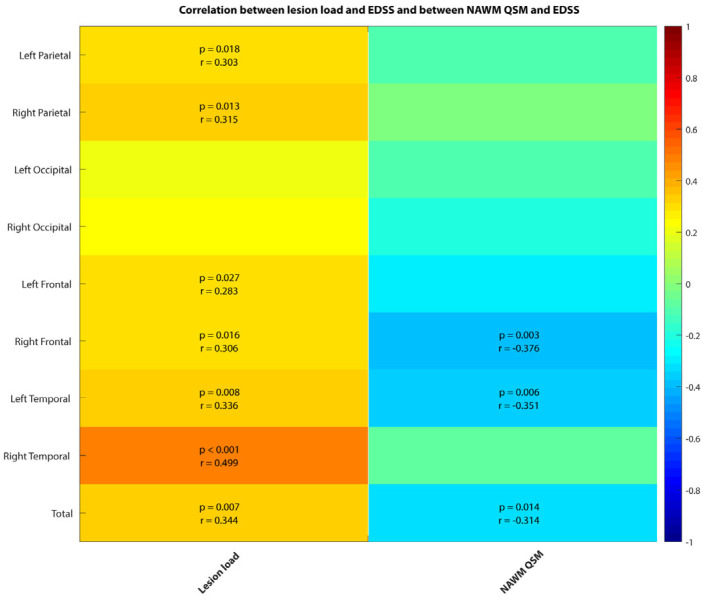
Correlation between EDSS and lesion load (first column) and between EDSS and NAWM susceptibility (second column). Only correlation coefficients with significant *p*-values are written down, with threshold value corrected for multiple comparison.

We found moderate to strong positive correlation between lesion load and EDSS score (rho = [0.28 to 0.5], *p* ≤ 0.03) and a weak to moderate negative correlation between EDSS score and total NAWM susceptibility, as well as with both frontal and left temporal lobe NAWM (rho = [−0.29 to −0.38], *p* < 0.014) (Figure 5). There was no correlation between WM morphometric values, nor with DGM susceptibility and morphometric values.

## 4. Discussion

The present study investigated the feasibility of a fully automated method for MS patient assessment and to evaluate the interrelation between lesion load and DGM, as well as NAWM susceptibility and morphometry and the correlation with the EDSS clinical score. We found negative correlations between lesion load and right thalamus susceptibility, as well as between lesion load and NAWM susceptibility. Lesion load was also negatively correlated with DGM and WM morphometric values. Despite these interrelations, the EDSS score only correlated with lesion load and anti-correlated with NAWM susceptibility values. Overall, the significant correlations and the tendencies to correlation obtained in our study were in line with a recent meta-analysis synthetizing data available on QSM mapping of MS lesions, DGM and NAWM. As the relation between lesion load and clinical disability has been often demonstrated, a significant relation with NAWM susceptibility values has almost never been obtained [34,35].

From the earliest description of lesions on MRI of MS patients, lesion load has been considered a biomarker of disease severity. However, numerous studies have reported that lesion load does in fact not perfectly correlate with patients’ disability [1,36,37]. Being related both to myelination and iron deposition, QSM was thought to have the potential for evaluating both lesions’ characteristics [4,38,39,40,41] and remote tissues changes, which could better reflect disease progression. We hereby demonstrated that a fully automated method for lesion detection and morphometric and QSM map computation is feasible in 93.2% of patients at 10 min per patient. This allowed for evaluating potential interrelations between lesion load, morphometry and susceptibility.

Indeed, positive correlations between lesion load and/or clinical disability and basal ganglia susceptibility were reported in the literature [10,42]. However, the relation between thalamic susceptibility and lesion load is still debated. Some studies showed positive correlations [43,44,45,46] and others found negative correlations, as in the present study [10,47,48,49]. Various reasons can explain these discrepancies, such as the type of QSM sequence (notably the number of employed echoes), post-processing steps (notably the type of normalization and referencing) and the subjects’ characteristics, which differ between studies. For instance, Chiang et al. [43] used a circular ROI in CSF for reference value, used manual segmentation of the lesions and did not include primary progressive multiple sclerosis patients. In our cohort, we used a double-echo SWI sequence, an in-house post-processing pipeline and referencing to the brain cortex that were all previously validated. Also, lesion load and DGM segmentation was performed using two automated prototypes that demonstrated good reproducibility [22,23,24,25] and ensured avoiding reader-related bias. Beyond technical considerations, this may be due to the heterogeneous evolution of susceptibility within the thalamus, as Schweser et al. [6] demonstrated that susceptibility reduction was more significantly associated with disease duration in the pulvinar, the left lateral nuclear region and the global thalamus. The discrepant results on thalamus susceptibility could also be due to the effect of atrophy, as Schweser et al. [19] recently suggested that magnetic susceptibility changes over time could be partially explained by disease-related atrophy, along with a progressive declining in DGM iron content. This argues for the necessity of analysing atrophy and susceptibility simultaneously, as in our study. In accordance, we found positive significant correlations between thalamus susceptibility values and thalamus, putamen and pallidum morphometry, which suggests a parallel evolution between DGM atrophy and iron loss. Regarding the morphometry, we also found a strong correlation between atrophy of DGM structures and lesion load (i.e., a negative correlation), which corroborates previous results, especially for the thalamus [12,16,18]. Deep grey matter atrophy, specifically in the thalamus and the basal ganglia, is considered to be a stronger and earlier biomarker of MS burden than lesion load [12,13,14,16,50]. Nevertheless, our results indicate that lesion load, DGM atrophy and susceptibility are interrelated markers of disease severity.

Beyond the DGM, recent studies evaluated the impact of MS on the surrounding NAWM. We here found a strong negative correlation between the lesion load and NAWM susceptibility, which is in line with evidence found in other studies [7,9,51], and a negative correlation between NAWM and EDSS, which is in line with a recent study [52]. Furthermore, we found that NAWM susceptibility decrease was correlated with thalamus susceptibility decrease and putamen susceptibility increase, in accordance with reported longitudinal iron level changes in MS patients [47]. Considering the prognostic value of early changes in the thalamus, this may sustain the influence of NAWM alteration on patients’ outcomes, as outlined by a longitudinal-exploring prediction value of NAWM susceptibility on EDSS increments over time [35]. In histopathological studies, the NAWM in MS patients has indeed shown substantial abnormalities, including inflammation, microglial activation, gliosis, demyelination and axonal swelling [53]. There is also evidence of decreasing iron content in NAWM with disease duration in chronic MS. This loss would contribute in a greater extent to NAWM changes on QSM than the concurrent myelin loss, which would lead to an increasing effect on QSM [9,51,54]. The decrease in susceptibility of the NAWM with increasing white matter lesions is thus likely due to a decrease of the iron/myelin ratio. However, Wang et al. [55] also reported that demyelination was significantly correlated with longitudinal atrophy of the NAWM. Along those lines, we found that the lesion load was negatively correlated with the WM morphometry in multiple lobes, which confirms the presence of remote changes in the brain of MS patients. In fact, while both iron loss and demyelination are associated with NAWM atrophy, their antagonist effect on susceptibility may explain discordant interrelations in the literature. This may also explain why we only observed a moderate association between NAWM susceptibility and WM morphometry. Overall, this suggests that iron loss, demyelination and atrophy of the NAWM are, as DGM changes, interrelated markers of disease progression and patients’ disability [55].

We have to acknowledge several limitations of the present study. First, there is increasing evidence that the underlying pathological processes of primary and secondary progressive MS are different, and this difference could impact advance MRI methods such as QSM [14,42,48,56]. In addition, the size of our patient cohort was too small to allow subgroup analysis, which is a meaningful analysis, given for instance the decreasing iron content within the thalamus from 30 years of age [57], or the influence of sex and genes involved in iron regulation on DGM susceptibility [49]. Also, our imaging protocol did not include diffusion [48] or myelin water imaging [58], which could help in evaluating microstructural changes and help characterizing the relative effect of iron loss and demyelination on susceptibility of the NAWM. While evaluating NAWM using susceptibility values or other techniques such as diffusion tensor imaging, neurite orientation dispersion and density imaging [59,60], T1/T2-weighted ratio [61] or MR spectroscopy [62] seems to provide new insights into physiopathological aspects of MS, it remains unclear which sequence or combination of sequences could have a favorable diagnostic or prognostic impact for initial patient classification, therapy guidance and monitoring or clinical outcome stratification. Exploring these points, as well as externally validating automated susceptibility quantification, needs larger prospective studies. The potential confounding effect of spinal cord lesions, trophicity and susceptibility on the interrelation with the EDSS could not be evaluated, as the spinal cord is not assessable with automated morphometry and QSM for its full length. Finally, longitudinal analysis of DGM and NAWM susceptibility and morphometry was not performed. This would need long-term follow-up and a larger, different cohort study. DGM atrophy [14] and DGM susceptibility [49] longitudinal evolution were already assessed in large studies to avoid the limitations of monocentric studies with short follow-up [47], as was evaluated NAWM susceptibility for clinical evolution prediction among different types of MS patients [35]. This could also help in evaluating whether different treatments might influence DGM and NAWM trophicity and susceptibility in a distinct manner, which has never been studied, to the best of our knowledge. To this purpose, integrated automated MR post-processing could be used, as in the present study.

In conclusion, automated computation of lesion load, morphometry and susceptibility maps is feasible and could allow the use of this advanced quantitative imaging in daily clinical practice. Lesion load, thalamus and NAWM quantitative susceptibility values and trophicity are interrelated in MS patients, as well, EDSS clinical score correlates with lesion load and anti-correlates with NAWM quantitative susceptibility. Beyond MS lesions, brain remote changes are potential biomarkers for disease monitoring and may be assessed using automated MR morphometry and QSM.

## Figures and Tables

**Table 1:** Imaging protocol.

Parameters	MP-RAGE Pre-Gd	3D FLAIR 3D	Double-Echo GRE
Voxel size	1 × 1 × 1.2 mm^3^	0.5 × 0.5 × 1 mm^3^	0.98 × 0.98 × 1.5 mm^3^
Acquisition plane	Sagittal	Sagittal	Transversal
Flip angle	9°	120°	15°
TR/TI	2300/900 ms	5000/1800 ms	46/-
Echo Time	2.9 ms	391 ms	20/40 ms

**Table 2:** Number of patients receiving each treatment at the time of the study.

Treatment	Patients
Tysabri^®^ (natalizumab)	17
Gilenya^®^ (fingolimod)	13
Tecfidera^®^ (dimethyl fumarate)	9
Rebif^®^ (interferon beta-1a)	4
MabThera (rituximab)	2
Plegridy^®^ (peginterferon beta-1a)	1
Avonex^®^ (interferon beta-1a)	2
Copaxone^®^ (glatiramer acetate)	2
No treatment	11

**Table 3:** Susceptibility values and morphometry of thalamus, DGM and WM (WM susceptibility values were computed after MS lesions exclusion).

	Susceptibility Value Median [IQR]		Z-Score Median [IQR]	
	Left	Right	Left	Right
Thalamus	0.0014 [0.0027]	0.007 [0.0081]	−0.1678 [1.8954]	−0.1454 [1.688]
Caudate nucleus	0.024 [0.0198]	0.027 [0.020]	−0.0915 [1.2053]	0.0878 [1.2287]
Putamen	0.0168 [0.0095]	0.0148 [0.008]	−0.0235 [1.2487]	−0.1012 [1.7689]
Pallidum	0.0573 [0.0152]	0.0607 [0.0157]	−0.1587 [1.5389]	−0.2118 [1.6012]
Frontal WM	−0.0033 [0.0031]	−0.0042 [0.0026]	−0.3771 [1.7347]	−0.3712 [1.3869]
Temporal WM	−0.0019 [0.0028]	−0.0037 [0.0041]	−0.4512 [1.5931]	−0.765 [1.7001]
Parietal WM	−0.0047 [0.0022]	−0.0054 [0.0029]	−0.7124 [1.4574]	−0.4795 [1.2212]
Occipital WM	−0.095 [0.0039]	−0.0083 [0.0052]	−0.1345 [1.0422]	0.1548 [1.2986]

## Data Availability

All the data can be obtained by contacting the corresponding author.
